# Development of *Acanthocheilonema viteae* in *Meriones shawi*: Absence of microfilariae and production of active ES‐62

**DOI:** 10.1111/pim.12803

**Published:** 2020-11-10

**Authors:** Felicity E. Lumb, James Doonan, Marlene Corbet, Miguel A. Pineda, Margaret M. Harnett, William Harnett

**Affiliations:** ^1^ Strathclyde Institute of Pharmacy and Biomedical Sciences University of Strathclyde Glasgow UK; ^2^ Institute of Infection, Immunity and Inflammation University of Glasgow Glasgow UK

**Keywords:** ES‐62, filarial nematode, jird, microfilaria, myeloid cell, osteoclast

## Abstract

**Aims:**

ES‐62 is a well‐studied anti‐inflammatory molecule secreted by L4‐adult stage *Acanthocheilonema viteae*. We maintain the life cycle of *A viteae* using *Meriones libycus* as the definitive host. Here, we investigated whether the full life cycle could be maintained, and functional ES‐62 produced, in a related jird species—*Meriones shawi*.

**Methods and Results:**

Adult worms were produced in comparable numbers in the two species, but very few microfilariae (MF) were observed in the *M shawi* bloodstream. *M shawi* ES‐62 produced ex vivo was functional and protective in a mouse model of arthritis. Myeloid‐derived cells from naïve and infected jirds of both species were compared with respect to ROS production and osteoclast generation, and some differences between the two species in both the absence and presence of infection were observed.

**Conclusions:**

The life cycle of *A viteae* cannot be successfully completed in *M shawi* jirds but L3 stage worms develop to adulthood and produce functional ES‐62. Preliminary investigation into jird immune responses suggests that infection can differentially modulate myeloid responses in the two species. However, species‐specific reagents are required to understand the complex interplay between *A viteae* and its host and to explain the lack of circulating MF in infected *M shawi* jirds.

## 1 INTRODUCTION

Filarial nematodes are a group of arthropod‐transmitted parasites of vertebrates. Adult worms, depending on species, are found in various tissues and body cavities of their definitive hosts and produce microfilariae (MF) that circulate in the bloodstream or migrate through the tissues to enable transmission. Filarial nematodes often induce lifelong infections, due to their ability to modulate their host's immune response to promote their own survival while at the same time, limiting host pathology. This immunoregulation is thought to be driven by the secretion of bioactive molecules by the nematodes.^1^ One of the best characterized secreted molecules is ES‐62, a phosphorylcholine‐containing glycoprotein produced by *Acanthocheilonema viteae*. ES‐62 is secreted by the post‐infective larva life cycle stages of the worm and can be detected in the serum of infected *Meriones libycus* jirds.^2^, ^3^ ES‐62 has been demonstrated to modulate the activity of multiple immune system cells such as dendritic cells, macrophages, mast cells and B and T lymphocytes [reviewed in^4^]. As a consequence, ES‐62 has been found to protect against development of autoimmune diseases such as rheumatoid arthritis [RA;^5^] and systemic lupus erythematosus [SLE;^6^]; allergic diseases such as asthma^7^ and, more recently, obesity‐accelerated ageing,^8^ in mouse models.

We routinely maintain the full life cycle of *A viteae* in the jird, M *libycus*, and adult worms can be retrieved and cultured ex vivo to isolate and purify ES‐62. Despite being an excellent host for *A viteae*, our *M libycus* colony is difficult to maintain due to the absence of a commercial source for providing new members (the colony was transferred to Strathclyde from the National Institute for Medical Research, London, in 1991) resulting in a high degree of inbreeding that makes it challenging to produce healthy offspring. *A viteae* can also be developed in the more readily available closely related *Meriones unguiculatus*,^9^ but our previous use of this species resulted in production of female worms notably smaller than those developed in *M libycus* (results not shown). We therefore explored whether we could establish the life cycle in a further closely related readily available species of jird, *Meriones shawi*, and whether nematodes from these jirds could produce functional ES‐62. We were also interested in investigating the effect of ES‐62 on the jird immune response. Despite having extensively studied the effect of ES‐62 in mouse models of disease, we know very little about how the molecule interacts with the immune system of a receptive host. Work in this area has been hampered by the lack of jird‐specific reagents required to investigate the effect of ES‐62 on specific immune cell populations; however, we were able to study the effects of the nematode product on certain functional responses of host cells.

## 2 METHODS

### 2.1 *Acanthocheilonema viteae* life cycle

The *Acanthocheilonema viteae* life cycle was maintained in *Meriones libycus* and in soft ticks (*Ornithodoros moubata*), as described previously.^10^ Briefly, adult male jirds were infected subcutaneously with 120 arthropod‐derived larvae (L3) which matured into adult worms over the course of 3 months. The number of MF was then determined in a small blood sample (5 µL), and jirds with an appropriate level of MF (approximately 120 MF in 5 µL blood) were used to infect ticks to complete the life cycle. Adult worms were obtained from jirds by dissection from under the skin of jird pelts following exsanguination using CO_2_ and counted and sexed—female worms were identified as larger nematodes while males are shorter and have a 'corkscrewed' phenotype. The worms were cultured ex vivo in 'complete' RPMI 1640 medium (containing 50 U/mL penicillin and 50 µg/mL streptomycin, supplemented with 10% glucose [Life Technologies]) for generation of ES‐62 as described previously.^5^, ^6^, ^7^, ^8^ The same procedures were applied to *Meriones shawi*.

### 2.2 In vitro macrophage differentiation

Bone marrow (BM) cells were collected from uninfected ('naïve') or infected *M libycus* or *M shawi* and cultured in 'complete' DMEM medium (containing 50 U/mL penicillin, 50 µg/mL streptomycin and 10% FCS, supplemented with 20% cell supernatant from the M‐CSF producing cell line L929). Fresh medium containing 20% L929 cell supernatant was added on day 4, and derived macrophages were used in experiments on day 7. BM‐derived macrophages (BMMs) were scraped in 'complete' RPMI 1640 medium, plated into 96‐well tissue culture plates, left to adhere overnight and then stimulated with *Salmonella enterica* LPS (0.1 µg/mL) or CpG‐ODN1826 (5 µmol/L) for 24 hours for functional assays.

### 2.3 Reactive oxygen species (ROS) flow cytometry assay

The presence of ROS in BM monocytes and BMMs was measured using 2′,7′‐dichlorofluorescein diacetate (DCF‐DA, Sigma). Briefly, ROS in BM monocytes was measured following red cell lysis, while ROS in BMMs was measured after stimulation with pathogen‐associated molecular pattern (PAMP) molecules for 24 hours. Cells were incubated with 50 µmol/L DCF‐DA for 30 minutes at 37°C and then washed in Fluorescence‐activated cell sorter (FACS) buffer (2.5% BSA; 0.5mM EDTA, in PBS) to stop the reaction. Data were acquired using a FACS Canto flow cytometer and analysed using FlowJo Software (Tree Star Inc, OR, USA, version 8.8.7). Populations were gated using non‐DCF‐DA stained controls.

### 2.4 In vitro osteoclast differentiation

Osteoclasts (OCs) were differentiated from naïve or infected *M libycus* or *M shawi* BM as previously described using mouse M‐CSF and RANKL reagents.^10^ BM was assessed for OC differentiation by TRAP staining (Leukocyte Acid Phosphatase Kit, Sigma) on day 6, and cells that stained positive for TRAP with ≥3 nuclei were counted as OCs. Images were obtained on an EVOS FL Auto Cell Imaging System at 20× magnification with scale bars set at 200 µm.

### 2.5 Collagen‐induced arthritis model

Male DBA/1 mice (8‐10 weeks; Harlan Olac), for the collagen‐induced arthritis (CIA) model,^5^, ^11^ were maintained at the Central Research Facility at the University of Glasgow (UoG) in accordance with the UK Home Office Licence PIL ICEBDB864 and PPL 60/4300 and the Ethics Review Board of the UoG. CIA was induced by intradermal immunization of mice with bovine Collagen Type II (CII) emulsified with complete Freud's adjuvant (MD Biosciences) on day 0, and animals challenged with CII in PBS (intraperitoneal injection) on day 21. Mice were treated subcutaneously with PBS or *M shawi* purified, endotoxin‐free ES‐62 (2 µg/dose) on days −2, 0 and 21. Joint damage (articular score) was scored as previously described.^5^, ^11^ Draining lymph nodes were dissected at cull and processed and analysed by flow cytometry based on size and granularity. Lymphocytes were categorized as either resting (small size and nongranular) or activated (small size and granular), and the percentage of activated cells within the lymphocyte gate was calculated.

### 2.6 Statistics

All data were analysed using GraphPad Prism 6 software, employing statistical analysis by Student's *t* test, one‐way ANOVA with Fisher's LSD post‐test, two‐way ANOVA with Tukey's multiple comparisons and linear regressions where appropriate. In all cases, *P < .05, **P < .01 and ***P < .001.

## 3 RESULTS

### 3.1 *Acanthocheilonema viteae* fecundity is host sub‐species specific

**FIGURE 1:**
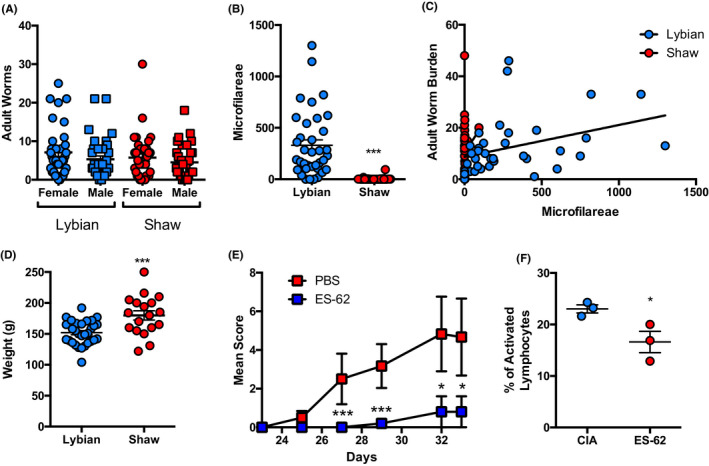
The *Acanthocheilonema viteae* life cycle cannot be completed in *Meriones shawi*. Age‐matched male *M libycus* and *M shawi* jirds were infected with 120 L3 *A viteae* larvae by sub‐cutaneous injection. Adult worms were dissected from jird pelts following exsanguination using CO_2_ and counted and sexed (A *M libycus*—n = 36; *M shawi*—n = 35). The presence of microfilariae (MF) was determined by blood sampling 5 µL and counting MF in each sample (B). Linear regression modelling was used to compare MF counts to adult worm burden, and the association of worm burden and MF count was confirmed in *M libycus* (*P* = .02) but not *M shawi* jirds (C). Infected *M libycus* (n = 31) and *M shawi* (n = 18) jirds were weighed (grams) after 3 mo of infection (D). ES‐62 purified from adult worms from *M shawi* jirds was tested for therapeutic properties in the mouse CIA model of arthritis with disease shown as mean arthritic score (ES‐62 n = 5; PBS n = 6; E). Draining lymph nodes from CIA and ES‐62‐treated CIA mice were analysed, and lymphocytes identified based on size and granularity (n = 3; F). Each data point represents an individual animal and is presented as mean ± SEM. Two‐tailed Student's *t* tests were used to analyse species differences, where * = *P* < .05 and *** = *P* < .001

*Acanthocheilonema viteae* is considered to only fully mature in a limited range of hosts of the rodent family Gerbillae, for example *M libycus* and *Gerbillus hirtipes*.^12^ We investigated whether a closely related species *M shawi* would be suitable as a definitive host for these parasites. Adult male *M libycus* and *M* *shawi* were infected with 120 L3 larvae derived from the tick intermediate host. Adult male and female worms were successfully obtained from both species with no significant difference in numbers (Figure 1A) or length of worms. However, the number of MF found in the blood of infected *M shawi* was significantly lower than the level in *M libycus* (Figure 1B), to the point of being essentially zero. Unlike some other filarial nematode species,^13^ we are not aware of periodicity having been described for MF of *A viteae*, but in any case examination of blood samples from infected *M shawi* at three different time points (~06.00, 11.00‐12.00 [our usual time] and 20.00‐22.00) over a number of days also did not lead to MF being detected. Moreover, examination of the peritoneal and pleural cavities of infected *M shawi* likewise failed to detect MF. Interestingly, in *M libycus* jirds the number of worms was significantly correlated with the number of MF, but this was not recapitulated in *M shawi* jirds (Figure 1C). Indeed, it was not possible to infect ticks from the latter species (results not shown) and so the full *A viteae* life cycle cannot be completed in *M shawi* jirds. Although these species are closely related, their significant difference in weight suggests that there may be a number of outward physical and perhaps physiological differences between these species (Figure 1D) but whether this has any impact on circulating MF numbers is unknown.

Similarly to adult worms from *M libycus*, worms from *M shawi* could be cultured in vitro for several weeks and comparable levels of ES‐62 purified from culture medium. The ES‐62 isolated from worms derived from *M shawi* and *M libycus* was compared on both denatured (SDS) and native PAGE gels and found to demonstrate the same molecular weights from the two species (data not shown). In order to determine whether the ES‐62 purified from adult worms from *M shawi* had similar immunomodulatory properties to ES‐62 obtained from *M libycus*, we tested the former in the CIA model of rheumatoid arthritis. As observed previously for *M libycus*‐derived ES‐62,^5^, ^11^ mice treated with *M shawi* ES‐62 were significantly protected against development of pathology in this model (Figure 1E). Additionally, treatment with *M shawi* ES‐62 was able to significantly suppress lymphocyte activation following ex vivo stimulation compared to control mice, confirming that *M shawi* ES‐62 was immunomodulatory in the model (Figure 1F).

### 3.2 *Acanthocheilonema viteae* trains host myeloid cell responses in *Meriones libycus*

**FIGURE 2:**
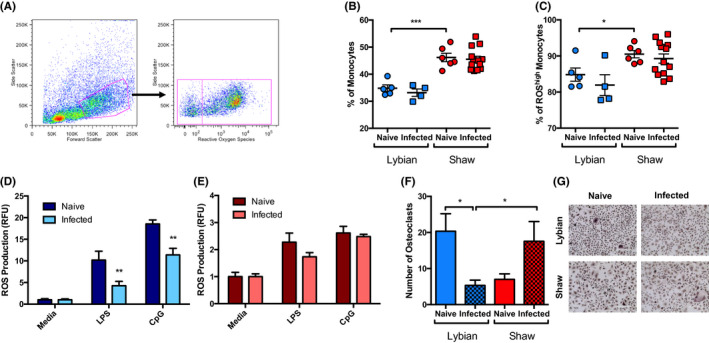
*Acanthocheilonema viteae* modulates host myeloid immune responses. Bone marrow cells from naïve or infected *M libycus* or *M shawi* jirds were collected at cull and subjected to red blood cell lysis and stained for the presence of ROS. Representative FACS plots of forward and side scatter identified a population of cells intermediate in granularity and large in size, reminiscent of monocytes that produce a high amount of ROS (A). The % of these monocyte‐like cells (B) and the % of ROS^high^ cells (C) were calculated. The data are combined from two experiments, and all data points reflect individual jirds with *M libycus* naïve, n = 5; *M libycus* infected, n = 4; *M shawi* naïve, n = 6; and *M shawi* infected, n = 13. BM‐derived macrophages (BMMS) were stimulated with 0.1 µg/mL of LPS or 5 µmol/L CpG, and ROS production after 24 h of stimulation was measured and normalized to medium controls for naïve and infected *M libycus* (D) and *M shawi* (E) jirds with each animal examined in triplicate. The data are combined from two experiments with *M libycus* naïve, n = 3 (9 technical replicates); *M libycus* infected, n = 3 (9 replicates); *M shawi* naïve, n = 3 (9 replicates); and *M shawi* infected, n = 6 (18 replicates). BM cells were also cultured in the presence of mouse M‐CSF and RANKL for 6 d and stained using TRAP; the numbers of multi‐nucleated TRAP + osteoclasts (F) and representative images of osteoclasts are shown (G; scale bar = 200 µm). The data are combined from three experiments with each animal examined in triplicate with *M libycus* naïve, n = 3 (9 technical replicates); *M libycus* infected, n = 5 (15 replicates); *M shawi* naïve, n = 3 (9 replicates); and *M shawi* infected, n = 6 (18 replicates). The data are presented as mean ± SEM and one‐ or two‐way ANOVAs with LSD Fisher's or Tukey's post‐tests were used to analyse species differences, where * = *P* < .05, ** = *P* < .01, and *** = *P* < .001

*Meriones shawi* adult worms were found to produce live MF when cultured ex vivo, and examination of these by light microscopy revealed no obvious difference from those produced by females developed in *M libycus*. Also, although we did not quantify MF release, examination of culture flasks indicated that the *M shawi* cultures contained MF levels which appeared to be no different to the (usual) high numbers present in the *M libycus* cultures. We therefore wished to investigate whether the low level of MF observed in the blood of *M shawi* was due to increased immune responses against the parasite. Due to a lack of species‐specific reagents, we have examined ubiquitous markers of inflammation as a preliminary attempt to measure aspects of the immune response to the nematode in *M shawi* and *M libycus*. First, we evaluated the level of intracellular ROS in BM cells from both species of jird using flow cytometry. We identified a monocyte‐like population of large cells with an intermediate granularity that produced ROS (Figure 2A) and found a significantly higher percentage of these cells in BM from *M shawi* compared to *M libycus* (Figure 2B). When we separated these cells based on expression of intracellular ROS, we observed that monocytes from *M shawi* have increased percentage of ROS^high^ cells compared to *M libycus* although this only reached statistical significance between naïve, but not infected, animals (Figure 2C). Infection did not impact on the percentage of these cell populations in either jird species (Figure 2B and C). Thus, to try and further address the effect of *A viteae* on host immune responses we differentiated BM cells from naïve and infected *M shawi* and *M libycus* jirds into macrophages (BMMs) and measured their ROS production after stimulation with PAMPs. Previously we have shown that in vivo treatment with ES‐62 can modulate in vitro host (mouse) BMMs by reducing pro‐inflammatory cytokine expression in response to PAMPs.^14^ While we were not able to detect cytokine production by jird BMMs due to lack of reagents, we found that infection of *M libycus* significantly and stably decreased the subsequent ROS production by BMMs when stimulated with LPS or CpG (Figure 2D). In contrast, infection of *M shawi* did not significantly inhibit these responses; however, this may be influenced by a poor response to the PAMPs compared to *M libycus* (Figure 2E). Of note, NO production at baseline and in response to PAMP stimulation could not be detected in jird BMMs (data not shown) highlighting an interesting immunological difference between jirds and more commonly used laboratory animals such as mice. We next investigated whether osteoclast (OC) differentiation was altered by helminth infection in these jirds as OCs are myeloid‐derived BM cells that rely on high ROS levels for differentiation and survival and which ES‐62 targets in its protection against joint disease.^11^ OC differentiation relies on both the pool of progenitors present in the BM and their responsiveness to stimulation. BM from the two jird species was cultured with mouse M‐CSF and RANKL for 6 days to promote their differentiation into OCs. As seen in Figure 2F and G, differentiation occurred more effectively with cells derived from *M libycus* and infection in this species significantly decreases the number of OCs. Conversely, infection in *M shawi* increases the number of OCs differentiated.

## 4 DISCUSSION

*Meriones shawi* was found to be an appropriate host for production of adult *A viteae* and subsequently, for generation of ES‐62 that was found to be biologically active. Despite the presence of viable adults, infected *M shawi* jirds had very low levels of circulating MF in their bloodstream, meaning the full nematode life cycle could not be completed in this species. There are a number of potential explanations for the lack of MF despite the development of viable adult worms: that the MF are being targeted by the immune response of the *M shawi* jirds, that the female adults are unable to produce MF, that the MF are located in a different anatomical site or that MF only periodically enter the bloodstream. On checking, we could find no clear evidence for the last two options and as living MF were observed in the medium when adult worms from *M shawi* were cultured ex vivo and that were not apparently different either quantitatively or qualitatively from MF released by females derived from *M libycus* when examined by light microscopy, there is clearly not a problem with fecundity.

With respect to immune responses, we did not attempt to measure antibodies against MF due to lack of reagents: however, previous studies in the model mouse host have shown that *A viteae* MF may be targeted by them.^15^ Nevertheless, interestingly, we found that *M shawi* jirds have a greater percentage of ROS^high^ monocytes than *M libycus* jirds suggesting that some immune responses may differ between the two species. However, infection had no effect on the percentage of such cells in either species. Nonetheless, infection with *A viteae* significantly reduced the production of ROS in response to bacterial PAMPs in BMM from *M libycus*, but not *M shawi*, jirds. Although the latter produce much lower levels of ROS when stimulated, possibly making differences more difficult to detect, this is a finding that may be worthy of further investigation relating to whether it impacts on MF numbers. Alternatively, it could be that the differences in myeloid responses observed in infected *M libycus* and *M shawi* may be influenced by the lack of circulating MF in *M shawi* rather than the infection per se. Recovery of cellular responsiveness in filariasis patients treated with microfilaricidal drugs such as DEC support the idea that MF play a key role in the modulation of the host immune response to the parasite. Indeed, stimulation in vitro with *Brugia malayi* MF lysate induced a regulatory phenotype in monocytes and macrophages characterized by expression of IL‐10 and PD‐L1.^16^ It is possible that the MF are reducing the potential for ROS generation in immune cells derived from *M libycus* as a mechanism to ensure their own survival. MF may be able to travel throughout the body including the BM^17^ and so could be having local effects in this 'organ', perhaps in some way accounting for the differences we see in osteoclast numbers differentiated from BM between jird species.

Any biological significance of the striking differential effect of infection on numbers of osteoclasts in the two jird species remains to be established and may be influenced by the relative ability of the two species—an observation that remains to be explored and explained—to differentiate osteoclasts in the absence of infection. These cells are primarily involved in maintenance of healthy bones but can adopt a pathogenic phenotype in diseases like rheumatoid arthritis. As mentioned earlier,^11^ ES‐62 can inhibit osteoclastogenesis in protecting against joint disease in the CIA model of RA, but the differential effect observed in the two jird species suggests it may not be active in this way in the present study, at least in *M shawi*.

## CONFLICT OF INTEREST

The authors have no conflicts of interest.

## AUTHOR CONTRIBUTIONS

JD and FL contributed equally to this manuscript, maintained the *Acanthocheilonema viteae* life cycle, and performed the experiments for the study that JD, FL and WH conceived. MC, MAP and MMH conceived, performed and analysed the mouse arthritis model. All authors were involved in reviewing and revising the manuscript and have approved the final version.

## DISCLOSURES

None.

## Data Availability

The data that support the findings of this study are available from the corresponding author upon reasonable request.
